# Forty-year study of rates of homicide by people with schizophrenia and other homicides in the Chuvash Republic of the Russian Federation

**DOI:** 10.1192/bjo.2021.1048

**Published:** 2021-12-01

**Authors:** Andrei Golenkov, Matthew Large, Olav Nielssen, Alla Tsymbalova

**Affiliations:** Chuvash State University, Cheboksary, Russia; Prince of Wales Hospital and University of New South Wales, Australia; Faculty of Medicine and Health Sciences, Macquarie University, Australia; Department of Judicial-Psychiatric Examination, Republic Psychiatric Hospital, Cheboksary, Russia

**Keywords:** Homicide, schizophrenia, alcohol, criminal responsibility, Russia (Chuvashia)

## Abstract

**Background:**

The extent to which rates of homicide by people with or without schizophrenia vary over time has theoretical and practical implications in understanding homicide by people with mental illness.

**Aims:**

The aim was to report on the rates of homicide by people diagnosed with schizophrenia over time in a region in which there were dramatic changes in the overall rates of homicide.

**Methods:**

An examination of homicide by people diagnosed with schizophrenia in the course of judicial psychiatric examination, and the rate of other homicide in the Chuvash Republic of the Russian Federation between 1981 and 2020 was undertaken.

**Results:**

During the 40 years of the study a total of 5741 people faced legal proceedings for a homicide offence, of whom 179 (3.1%) were diagnosed with schizophrenia. During the study period the average annual total homicide rate rose from about 9 per 100 000 in the 1980s, peaked at 17 per 100 000 in the 1990s before falling to 13 per 100 000 in the 2000s and 6 per 100 000 in the 2010s. Rates of homicide by people with schizophrenia also rose and fell over this period and were significantly associated with the rates of other homicide (*r* = 0.503, d.f. = 38, *P* = 0.001).

**Conclusions:**

The rise and fall in rates of homicide by people diagnosed with schizophrenia in parallel to total homicide suggests that homicidal behaviour might not be intrinsic to the clinical manifestations of the illness, and might instead reflect a heightened vulnerability to social factors that are associated with homicide by people without schizophrenia.

## Background

There is a well-recognised but poorly understood association between schizophrenia and homicide offending. Meta-analyses of available studies show a fourfold increased risk of homicide offending among men with schizophrenia and an eightfold increased risk among women compared with rates of homicide offending by people without schizophrenia,^1^ and that a pooled estimate of 6.5% of homicide offenders have schizophrenia.^2^ The traditional view has been that the elevated homicide risk among people with schizophrenia is because homicide risk is intrinsic to clinical manifestations of schizophrenia, particularly persecutory delusional beliefs.^3^ This view has contributed to an emphasis on the treatment of psychotic symptoms and on measures to contain people with schizophrenia who are perceived as potentially violent.^3^ This view was supported by studies suggesting that homicide rates by people with severe mental illness would be more or less fixed over time and between regions, in line with the stability in prevalence of severe mental illness.^4,5^ However, a 2009 meta-analysis of 18 available population-based studies found that rates of homicide by people with schizophrenia are heterogeneous and are strongly correlated to rates of other homicide. This study contradicts earlier views about the stability and causes of rates of homicide by people with schizophrenia and suggests that other explanations, including the possibility that rather than being intrinsic to the psychopathology of schizophrenia, people with schizophrenia might be more vulnerable to the same risk factors that determine homicide rates in the general community.^2^ The implications of this finding include that homicide by people with schizophrenia might best be prevented by limiting the exposure to factors known to be associated with violence in the community, such as access to firearms and limiting alcohol and other drug use, as much as by treatment and hospital admission. One limitation to the 2009 meta-analysis^2^ was that it examined aggregated homicide rates over the duration of the included studies and did not examine changes in rates or in the relationships between the rates of homicide by people with and without schizophrenia over time.

Several studies have examined the trends in homicide committed by a broader class of people considered to have a mental disorder. In England and Wales, rates of homicide by people with mental illness (defined as the total of those judged to have diminished responsibility, infanticide, or as being unfit to stand trial or not guilty due to mental illness) rose steeply with rates of other homicide after World War Two, before falling just as steeply, while the rate of other homicides continued to rise.^6^ Another pattern was observed in New Zealand between 1970 and 2000 where the total homicide rate rose annually between 1970 and 1990 before falling in the last decade of the study, whereas the proportion of homicide by people defined as ‘mentally abnormal’ (not guilty due to mental illness, infanticide and those unfit to stand trial) fell throughout the study, initially because homicide by people who were not mentally ill was rising and later because mental illness verdicts were falling faster than other court outcomes.^7^

In Denmark, homicide by people with psychosis (defined using ICD-8 criteria) were reported to be stable during a period of rising homicide rates between 1956 and 1970, but then increased in parallel with total homicide rates between 1970 and the mid-1980s.^8^ In Iceland homicide by people with mental illness (defined as schizophrenia, manic depression and morbid jealousy) rose more quickly than rapidly rising rates of other intentional homicides in the period 1970 to 1984.^9^ More recently, a study from Ontario, Canada found a non-significant fall in homicide offending by people who were mentally ill during a period when the rate of other homicide fell more.^10^ To date the only study that focused on changes in rates of homicide by people with schizophrenia found a rise in the absolute number of homicide offenders with schizophrenia during a period in which other homicide rates fell in England and Wales.^11^ The major limitation in interpreting this group of studies as an indicator of the association between homicide offending by people who are mentally ill compared with those who are not mentally ill is the differences between studies in the way mental illness is diagnosed and defined in law between jurisdictions and over time.^2,12^

## Aims

We have previously reported on the characteristics of offenders with schizophrenia and other mental disorders from The Chuvash Republic of the Russian Federation, a region with both a high total homicide rate and a high rate of homicide by people with schizophrenia.^13–15^ Previously we found that almost half of all homicide offenders had at least one mental disorder, including alcohol dependence experienced by 16% of offenders and schizophrenia experienced by over 4% of offenders.^15^ In this paper we report on the trends in rates of homicide by people with schizophrenia and without schizophrenia (other homicides) during a period of 40 years when the rate of total homicide rose and fell dramatically. We hypothesised that if rates of homicide by people with schizophrenia were fixed over time that they would not be correlated with the rates of homicide by people without schizophrenia and that the proportion of homicides by people with schizophrenia would fall while other homicide rates were rising and would rise when other homicide rates were falling. In other words, the rate of homicide by people with schizophrenia did not change when the overall rate of homicide changed .

## Method

### Sample

The study examined a data-set of all homicides in Chuvash Republic of the Russian Federation between January 1981 and December 2020. The Chuvash Republic has a population of over 1.2 million living in an area of around 18 000 km^2^, centred in the city of Cheboksary on the Volga River about 650 km east of Moscow. A third of the population live in Cheboksary and 40% are considered to be rural. During the period of the study, and as in the rest of Russia, people in the Chuvash Republic who are charged with a homicide offence are referred for psychiatric evaluation under Section 21 of the Russian Penal Code.^13–16^ Referral for judicial psychiatric examination by a panel of three psychiatrists is at the discretion of the investigators and the court. Some accused are not referred because there are no doubts about their sanity, and some individuals who are accused refused to be examined. However, most accused are examined, and the sample includes the majority of offenders referred for a formal evaluation, including all offenders suspected of having a psychiatric disorder. Data was also available about homicides that were immediately followed by the suicide of offenders known to mental health services.

### Case identification and data extraction

Demographic, clinical and criminological data were extracted from the case files, which included the judicial psychiatric report, hospital records, out-patient notes and legal documents. About one-third of the offenders had been personally examined by either A.G. or A.T. The demographic data extracted were age, gender, occupation, marital status and years of education and the clinical data included psychiatric diagnosis, age at onset, previous treatment, pattern of symptoms, history of brain injury, and substance misuse, and the criminological data included previous convictions, method of homicide and judicial outcome. The psychiatric diagnoses were made using the version of the ICD that was current at the time of the judicial examinations. For the purpose of this study the psychiatric diagnoses in each individual was made by retrospective chart review using ICD-10 criteria.^17^

### Statistical analysis

Non-age-adjusted homicide rates were calculated using the number of annual homicide incidents and the annual population of Chuvashia, as measured by the census or imputed using linear assumptions in the intervening years. The association between rates of homicide by people with schizophrenia and other homicide and the association between the rates of other homicide and the proportion of all homicides committed by people with schizophrenia were examined with a Pearson correlation coefficient using untransformed homicide rates.

The changes in homicide rates by people with and without schizophrenia and changes in the proportion of homicides (homicides by people with schizophrenia/total homicides) committed by people with schizophrenia over time were examined using Kendall's Tau rank correlation coefficient over the whole period of the study and over two equal periods (1981–2000 and 2001–2020) in which the homicide rates were, respectively, rising and falling. In order to illustrate the association between homicide in schizophrenia and other homicide, rates were displayed as Log_10_ (1 + homicide rate per million population per annum) so as to avoid the scale minimisation effects that can arise because of the low base rate of homicide offending by people with mental illness.^18^ Possible differences in the characteristics of male and female homicide offenders with schizophrenia were examined using *t*-test and chi-square (χ^2^). When one or several cells contained five individuals or less, a Fisher's Exact test was used. All tests were performed in a two-tailed form. The data were analysed using SPSS Version 27 (SPSS Inc. Chicago, IL, USA).

### Ethical approval

Approval for the research was obtained from the Ethics Committee of the Medical Faculty of Chuvash State University, and the Chuvash Association of Psychiatrists, Narcologists, Psychotherapists and Medical Psychologists (a branch of the Russian Society of Psychiatrists). As the study only uses statistical information, no oral or written consent was taken from the individuals of the study.

## Results

### Rates of homicide

In the 40 years from the beginning of 1981 and the end of 2020, 5741 people faced legal proceedings for a homicide offence, and 3410 homicide offenders were referred for psychiatric evaluation. Over this period there were more homicide victims than offenders because the police did not solve every case, some offenders had multiple victims, and some offenders died by suicide or died prior to being examined. A small number of offenders from other parts of Russia were examined elsewhere. The homicide clearance rates, defined as an offender being identified by the police, were more than 90% in the period of the study. Of those subjected to judicial examination, 46.7% were found to have at least one mental disorder, and 7.7% were found to be not responsible for their actions because of mental disorder according to the Russian Criminal Code.^18^

During the study period 179 homicide offenders were identified as having schizophrenia, including 14 recidivist offenders who committed both homicides between 1981 and 2020 (Table 1). The population of Chuvashia rose slightly from 1.304 million in 1981 to 1.347 million in 1993, and subsequently fell by about 10% to 1.224 million in 2020.

**Table 1:** Homicide by people with schizophrenia in Chuvashia 1981–2020

	Homicide offenders with schizophrenia, *n*	Female homicide offenders with schizophrenia, *n*	Adolescents, *n* (age, years)	Recidivist homicide offence, *n* (year index)	Two victims or more, *n* (*n* of victims)	Total people with schizophrenia in Chuvashia, *n*	Total homicide in Chuvashia, *n*
1981	2			1 (1966)			113
1982	5				1 (4)		107
1983	3		1 (19)			3102	121
1984	3	1		1 (1965)			146
1985	1						106
1986	5					3182	84
1987	4				1 (3)		94
1988	7	1	1 (12)		1 (2), 1 (2)		118
1989	2						132
1990	6	3		1 (1978)			149
1991	1						127
1992	5			1 (1983)			194
1993	7	2		1 (1976)		4583	220
1994	7	1	1 (17)		1 (2)		238
1995	4		1 (19)				246
1996	4	1			1 (2)		210
1997	6				1 (2)	4491	233
1998	6			1 (1988)			223
1999	5	1		1 (1994)			257
2000	10	2					250
2001	2						229
2002	5	1	1 (18)				203
2003	10		1 (19), 1 (15)	1 (1988)	1 (2)		202
2004	5		1 (17)		1 (2)	4875	186
2005	6			1 (1990)			175
2006	8			1 (1999)	1 (4),1 (2)		153
2007	8		1 (19)	2 (1994), (1995)			128
2008	5		1 (19)	3 (1992, 1998, 2000)			129
2009	4						106
2010	7	1		2 (1987, 1997)	1 (2)	4812	102
2011	3^a^	1			1 (2)		99
2012	2	1			1 (2)		82
2013	4			1 (2003)			94
2014	1						93
2015	7^a^	3	1 (15)		1 (3)		101
2016	3						73
2017	2						86
2018	0					4747	42
2019	2					4659	46
2020	2					4736	44
Total	179	19	11	18^b^	15 (36)^c^		

a. Homicide–suicide known schizophrenia *n* = 2 (2011 – female, 2015 – male).

b. First homicide.

c. Victims.

During the four decades of the study the rates of homicide by people without schizophrenia rose and fell:

- 1981–1990: average 8.9 per 100 000 person-years;
- 1991–2000: average 16.4 per 100 000 person-years;
- 2001–2010: average 12.5 per 100 000 person-years;
  2011–2020: average 6.1 per 100 000 person-years.

Rates of homicide by people with schizophrenia also rose and fell in the same periods:

- 1981–1990: average 0.28 per 100 000 person-years;
- 1991–2000: average 0.41 per 100 000 person-years;
- 2001–2010: average 0.47 per 100 000 person-years;
- 2011–2020: average 0.21 per 100 000 person-years.

**Fig. 1:**
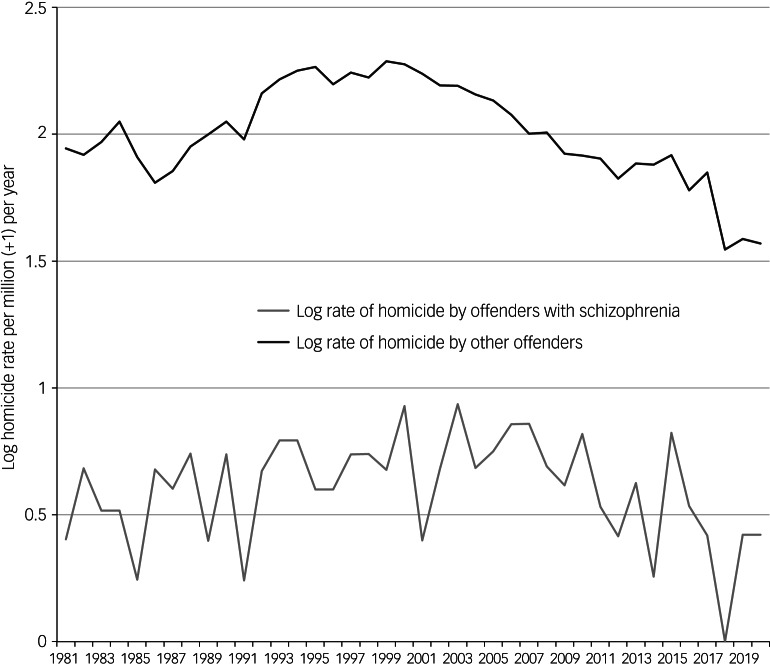
Homicide rates by people with schizophrenia and other homicide in Chuvashia 1981 to 2020.

The annual rate of homicide by people with schizophrenia was strongly correlated with the rate of other homicide (Pearson's *r* = 0.503, *r*^2^ = 0.253, *F* = 12.9, d.f. = 38, *P* = 0.001) (Fig. 1). There was no significant association between the rates of other homicide and the proportion of all homicides committed by people with schizophrenia (Pearson's *r* = −0.21, *r*^2^ = 0.045, *P* = 0.19).

In the period 1981–2000 rates of homicide by people with schizophrenia rose significantly (tau (τ) = 0.33 *P* = 0.04) as did those without schizophrenia (τ = .67 *P* = <0.0001). In the period 2001–2020 rates of homicide by people with schizophrenia fell significantly (τ = −0.35 *P* = 0.03) as did those by people without schizophrenia (τ = −0.86 *P* < 0.0001) (Fig. 1).

The proportion of homicides by people with schizophrenia did not change over the 40 years of the study (τ = 0.15, *P* = 0.18), during the period 1981–2000 when rates of homicide were rising (τ = −0.05 *P* = 0.75) or in the period 2001–2020 when rates of homicide were falling (τ = 0.06 *P* = 0.7)

### Characteristics of homicides by people with schizophrenia

In total 160 homicide incidents were committed by men with schizophrenia and 19 (10.7%) by women with schizophrenia. Adolescents (defined as individuals aged between 12 and 19 years) with schizophrenia committed 11 (6.2%) homicide offences (Table 1). There were 18 recidivist homicides by people with schizophrenia, 17 of which took place in rural areas, including 14 who committed their first and second homicide in the period of the study and 4 whose initial homicide was before 1981. There were 15 homicides with multiple victims, including 3 by women, and 2 homicides followed by suicide by people who were known to have schizophrenia. Thirty-one offenders were sentenced to indefinite hospital care, and the remainder received fixed terms (Table 2).

**Table 2:** Characteristics of homicide offenders with schizophrenia 1981–2020

	Total offenders	Female offenders	Male offenders	χ^2^ (d.f.)	*P*	Total, *n*	Female, *n*	Male, *n*
Age, years: mean (s.d.), range	36.84	(12.43)	37.73	(12.33), 24–70	36.84	(12.64) 12–63					
Comorbid traumatic brain injury, *n (%)*	41	(24.85)	1	(5.26)	40	(27.39)	5.675	0.018	165	19	146
Marital status, *n* (%)							9.737 (3)	0.020			
Single	99	(60.00)	9	(47.37)	90	(61.64)			165	19	146
Divorced	24	(14.55)	2	(10.53)	22	(15.07)			165	19	146
Widow	1	(0.60)	1	(5.26)	–	–			165	19	–
Married (civil marriage)	41	(24.85)	7	(36.85)	34	(23.29)			165	19	146
Location, rural, *n* (%)	93	(56.36)	10	(52.6)	83	(56.85)	0.353		165	19	146
Education, *n* (%)							8.496 (4)	0.075			
No formal education	2	(1.21)	1	(5.26)	1	(0.68)			165	19	146
Attended school	56	(33.94)	2	(10.53)	54	(37.00)			165	19	146
Completed school	78	(47.27)	13	(68.42)	65	(44.52)			165	19	146
College education	21	(12.73)	2	(10.53)	19	(13.01)			165	19	146
University education	8	(4.85)	1	(5.26)	7	(4.79)			165	19	146
Age at onset of schizophrenia, mean (s.d.)	22.1	(7.5)	24.57	(9.61), 15–55	22.52	7.30 (8–47)					
Duration of schizophrenia, mean (s.d.)	14.7	(10.8)	13.00	(11.25), 0.5–36	14.28	(10.71), 0.5–36					
Legal outcomes, *n* (%)											
Conviction	46	(27.88)	1	(5.26)	45	(30.82)	6.807	0.009	165	19	146
Treatment in a psychiatric hospital by court order	31	(18.79)	-	-	31	(21.23)	6.456	0.010	165	–	146
Suicidal behaviour	34	(20.61)	8	(42.11)	26	(17.81)	6.067	0.013	165	19	146
Previous criminal conviction	46	(27.88)	4	(21.10)	42	(28.77)	0.955		165	19	146
Method of homicide, *n* (%)							12.713 (6)	0.047			
Beaten	39	(21.79)	7	(36.85)	32	(20.00)			179	19	160
Stab–cut	93	(51.96)	9	(47.37)	84	(52.50)			179	19	160
Strangled	25	(13.97)	1	(5.26)	24	(15.00)			179	19	160
Firearm	2	(1.12)	–	–	2	(1.25)			179	–	160
Set fire	1	(0.55)	–	–	1	(0.63)			179	–	160
Threw from a height	1	(0.55)	1	(5.26)	–	–			179	19	–
Several ways	18	(10.06)	1	(5.26)	17	(10.62)			179	19	160
Victims and circumstances (*n* = 200), *n* (%)							35.990 (4)	<0.001			
Children^a^	17	(8.50)	9	(39.13)	8	(4.52)			–	23	177
Spouses (couple)	19	(9.50)	2	(8.70)	17	(9.60)			–	23	177
Relatives	78	(39.00)	10	(43.47)	68	(38.42)			–	23	177
Acquaintances (neighbours)^b^	79	(39.50)	2	(8.70)	77	(43.50)			–	23	177
Strangers	7	(3.50)	–	–	7	(3.96)			–	–	177
Two victims or more, *n* (%)	15	(8.38)	3	(15.79)	12	(7.50)	0.632		179	19	160
Alcohol intoxication, *n* (%)	83	(46.37)	1	(5.26)	82	(51.25)	16.351	<0.001	179	19	160
On the street, *n* (%)	35	(19.55)	2	(10.53)	33	(20.63)	1.836		179	19	160
Attempts to conceal, *n* (%)	21	(11.73)	1	(5.26)	20	(12.50)	1.699		179	19	160

a. Victims were children (39.13% v. 4.52% in males; d.f. = 1; χ^2^ = 31.351; *P* < 0.001).

b. Victims were acquaintances (neighbours) (43.50% v. 8.70% in females; d.f. = 1; χ^2^ = 8.914; *P* = 0.0028 as amended by Yeats).

## Discussion

### Main findings

This study examines the rates of homicide by people with schizophrenia over a period of 40 years in a region that had a dramatic rise and equally dramatic decline in the rate of homicide. The results confirm the well-recognised over-representation of people with schizophrenia among homicide offenders, but also suggest that the rate of homicide by those with schizophrenia is correlated with changes in the rate of other homicide and that the proportion of homicides committed by people with schizophrenia might not fall when other homicide rates are higher and might not rise as other homicides become less common.

### Interpretation of our findings

Although the reasons for the five- to tenfold increase in homicide risk associated with schizophrenia remains uncertain, the findings of this study suggest that the increased rate is at least partly because of a greater exposure or greater sensitivity of people with schizophrenia to more general risk factors for homicide, including factors such as substance use and social disadvantage, rather than an intrinsic or inevitable propensity to lethal violence associated with the illness.

In this study the *r*^2^ statistic indicated that about one-quarter of the variance in the rate of homicide associated with schizophrenia was explained by the changes in the overall rate of homicide. This can be compared with the results of a 2009 meta-analysis that found that three-quarters of the variance in rates of homicide in schizophrenia between jurisdictions was explained by the rates of other homicide.^2^ Hence both cross-sectional and longitudinal studies suggest that homicide rates by people with schizophrenia are not fixed over time and between regions and that social factors that influence homicide by people without schizophrenia, for example, alcohol and other drug use, levels of violence in the community, and the efficiency of the police in preventing crimes influence the likelihood of a person with schizophrenia committing a homicide offence. However, the moderate *R*^2^ value in the association between rates of homicide in people with schizophrenia and other homicide does suggest that there are other factors that influence the rate homicide in schizophrenia that operate independently to those associated with total homicide. These might include the availability and quality of psychiatric care, variation in the prevalence of schizophrenia, and the specific impact of substance use on people with schizophrenia.

The decline in the total rate of homicide in Chuvashia in the past 20 years is striking, and may be because of greater economic prosperity and the re-establishment of social order after the turbulent glasnost years. The decline may also be related to the ageing of the population, as many people of working age have moved elsewhere, and a rise in the proportion of women in the population. There was an increase in the number of people known to have schizophrenia in Chuvashia during the years of the study that might be as a result of an increase in the identification and reporting of patients with schizophrenia by a larger mental health workforce. Hence, it is also possible that an increase in availability of treatment contributed to the decline in homicide offences by people with schizophrenia, as suggested by the near elimination of recidivist homicide offences by people with schizophrenia in the past decade and the over-representation of recidivists in rural areas where services are less available. During the period of the study there were no changes to the laws governing psychiatric treatment, which has been proposed as a possible reason for changes in rates of homicide by people who are mentally ill in the USA, along with the quality of services.^19^

A decline in the consumption of alcohol in Chuvashia might also have been important. During the period of the study alcohol consumption in Chuvashia fell from being above the Russian national average to well below the national average^20^ during a period in which the national per capita alcohol consumption fell by more than 40%.^21^ About half of the offenders with schizophrenia (83/179, 46%) were reported to be intoxicated with alcohol at the time of the offence, a pattern that had not changed in the decade since an earlier report.^14^

### Strengths and limitations

Strengths of this study are the complete nature of the sample and related statistical information, consistency in the way the psychiatric examinations were recorded and an absence of changes to relevant sections of the legal code. Limitations of the study include that some homicide offenders did not receive a judicial examination, not all homicides were solved by the police, and the absence of information about the mental state of some of the homicide offenders who died by suicide before they could be examined.

### Implications

A more reliable estimate of the extent to which rates of homicide by people with schizophrenia vary from other homicide rates might be obtained by meta-analysis once further longitudinal studies have been conducted in other jurisdictions. The extent of this correlation has implications for our understanding of the reasons for homicide by people with schizophrenia, and the degree to which systems of treatment for psychosis, and more general measures to reduce violence and homicide in the community might play a role in the prevention of homicide by people with emerging and established mental illness.

## Data Availability

The complete data-set is held by A.G. and is available for research purposes on reasonable request and ethical review.
